# Loss of Mtm1 causes cholestatic liver disease in a model of X-linked myotubular myopathy

**DOI:** 10.1172/JCI166275

**Published:** 2023-09-15

**Authors:** Sophie Karolczak, Ashish R. Deshwar, Evangelina Aristegui, Binita M. Kamath, Michael W. Lawlor, Gaia Andreoletti, Jonathan Volpatti, Jillian L. Ellis, Chunyue Yin, James J. Dowling

**Affiliations:** 1 Program in Genetics and Genome Biology, The Hospital for Sick Children, Toronto, Ontario, Canada.; 2 Department of Molecular Genetics, The University of Toronto, Toronto, Ontario, Canada.; 3 Division of Clinical and Metabolic Genetics and; 4 Division of Gastroenterology, Hepatology and Nutrition, The Hospital for Sick Children, Toronto, Ontario, Canada.; 5 Medical College of Wisconsin, Milwaukee, Wisconsin, USA.; 6 Translational Science Laboratory, Milwaukee, Wisconsin, USA.; 7 Astellas Gene Therapies, San Francisco, California, USA.; 8 Division of Gastroenterology, Hepatology and Nutrition and Division of Developmental Biology and; 9 Center for Undiagnosed and Rare Liver Diseases, Cincinnati Children’s Hospital Medical Center, Cincinnati, Ohio, USA.; 10 Division of Neurology, The Hospital for Sick Children, Toronto, Ontario, Canada.

**Keywords:** Hepatology, Muscle Biology, Monogenic diseases

## Abstract

X-linked myotubular myopathy (XLMTM) is a fatal congenital disorder caused by mutations in the *MTM1* gene. Currently, there are no approved treatments, although AAV8-mediated gene transfer therapy has shown promise in animal models and preliminarily in patients. However, 4 patients with XLMTM treated with gene therapy have died from progressive liver failure, and hepatobiliary disease has now been recognized more broadly in association with XLMTM. In an attempt to understand whether loss of *MTM1* itself is associated with liver pathology, we have characterized what we believe to be a novel liver phenotype in a zebrafish model of this disease. Specifically, we found that loss-of-function mutations in *mtm1* led to severe liver abnormalities including impaired bile flux, structural abnormalities of the bile canaliculus, and improper endosome-mediated trafficking of canalicular transporters. Using a reporter-tagged Mtm1 zebrafish line, we established localization of Mtm1 in the liver in association with Rab11, a marker of recycling endosomes, and canalicular transport proteins and demonstrated that hepatocyte-specific reexpression of Mtm1 could rescue the cholestatic phenotype. Last, we completed a targeted chemical screen and found that Dynasore, a dynamin-2 inhibitor, was able to partially restore bile flow and transporter localization to the canalicular membrane. In summary, we demonstrate, for the first time to our knowledge, liver abnormalities that were directly caused by *MTM1* mutation in a preclinical model, thus establishing the critical framework for better understanding and comprehensive treatment of the human disease.

## Graphical abstract

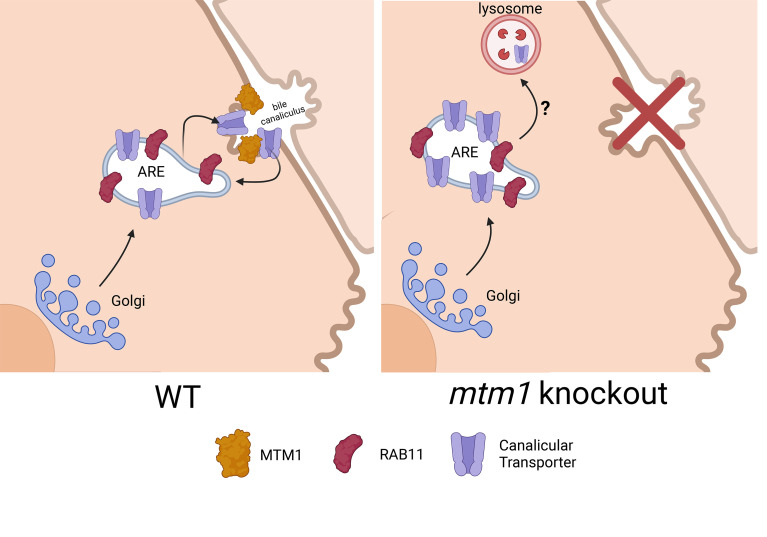

## Introduction

X-linked myotubular myopathy (XLMTM) is a rare congenital muscle disease characterized by severe skeletal muscle weakness and hypotonia and occurs in 1 of 50,000 male births (1–3). Approximately 25%–50% of patients with XLMTM die in the first year of life, and the surviving affected individuals have multiple technology dependencies (80% require tracheostomy and a feeding tube) and reduced lifespan (3). There are currently no approved treatments for this disease, although various therapies are in development (4). Of these, resamirigene bilparvovec (AT132), an AAV8-driven gene replacement therapy, has shown great promise in murine and canine models and entered clinical trial in 2017 for patients with XLMTM (ASPIRO, ClinicalTrials.gov NCT03199469) (5). Initial data have been promising with regard to restoration of muscle function; however, the trial has been halted because of the death of 4 study participants from liver failure associated with cholestasis (6, 7). Potential liver manifestations in XLMTM are poorly understood. Patients with XLMTM have been reported to exhibit signs of liver dysfunction including elevated liver enzymes and bilirubin (3). In addition, a recent case series reported features of intrahepatic cholestasis in 4 patients with XLMTM (8). The molecular mechanisms and etiology of cholestasis in patients with XLMTM remain unknown, and understanding the pathophysiology of this process will be critical for current patient care and for the development of therapeutics. Of note, liver pathology has yet to be described in any mammalian model of the disease.

XLMTM is caused by mutations in the myotubularin gene (*MTM1*). MTM1 encodes a phosphoinositide phosphatase that specifically targets the phosphoinositides PI(3)P and PI(3,5)P_2_ (9, 10). Phosphoinositides (PIs) are lipids found on membrane-bound organelles within the cell. Despite making up less than 1% of cellular lipids, they play important roles in diverse processes such as cell signaling and membrane trafficking (11). PI(3)P and PI(3,5)P_2_ are primarily localized to endosomes, where they act as docking sites to regulate the recruitment of effector proteins that control trafficking of protein cargo (11). Endosomal trafficking is critical in hepatocytes for targeting canalicular transporters to the apical membrane, as well as to respond to cues regulating increased bile transport (12).

The production and secretion of bile, a key function of the liver, is a complex process that is dependent on hepatocyte polarity. Bile secretion occurs at the canalicular membrane of hepatocytes and is governed by a set of bile transporter proteins (12). These proteins are critically important for bile secretion and are thus highly regulated at the protein level through subapical recycling endosomes (13). Dysregulation of these canalicular transporters can result in cholestasis, a disease state characterized by impairments in bile flow, causing intrahepatic accumulation of toxic bile acids (14). For example, mutations in *ABCB11*, which encodes bile salt export pump (BSEP), are associated with a genetically inherited form of cholestasis called progressive familial intrahepatic cholestasis type 2 (PFIC2) (12, 13).

Three primary animal models have been used to study XLMTM: a murine gene-knockout model developed using homologous recombination, a canine model of spontaneous mutation of MTM1, and a zebrafish model created using zinc finger nuclease–based gene editing (14–16). The murine and canine models do not show apparent signs of liver dysfunction, whereas the preliminary description of the zebrafish model noted increased liver size (17). To our knowledge, the extent and nature of liver dysfunction in the zebrafish model have not previously been studied, nor have the underlying mechanisms been explored. The zebrafish, given its optical clarity and cellular and genetic similarity to humans, provides an ideal opportunity to examine liver pathology in the context of *MTM1* deficiency (18–20). Zebrafish have previously been shown to be a faithful model of liver disease caused by a variety of genetic and environmental factors, including recapitulation of changes seen in the liver with BSEP deficiency (13, 18).

In this study, we provide an in-depth characterization of the liver phenotype of the zebrafish model of XLMTM. We identify changes in the structure of the bile canaliculus and disturbances in bile flux, both of which are consistent with cholestatic liver disease. We determine that canalicular transport protein localization and expression were altered, which likely accounts for the underlying cholestatic phenotype. Expression of exogenous Mtm1 in the liver revealed localization to a complex containing both Rab11 and canalicular transport proteins, consistent with a role in endosomal sorting. This expression was sufficient to rescue the cholestatic phenotype, confirming that it was liver autonomous and due to the loss of *mtm1* expression. Finally, we performed a targeted chemical screen and identified a dynamin-2 (DNM2) chemical inhibitor that could rescue the changes in canalicular structure and function. In all, we provide what we believe to be the first demonstration of a direct role for MTM1 in the liver and provide the critical groundwork for understanding and treating the liver phenotype recently uncovered in patients with XLMTM.

## Results

### mtm zebrafish have abnormal bile flux and accumulation of bile salts.

**Figure 1:**
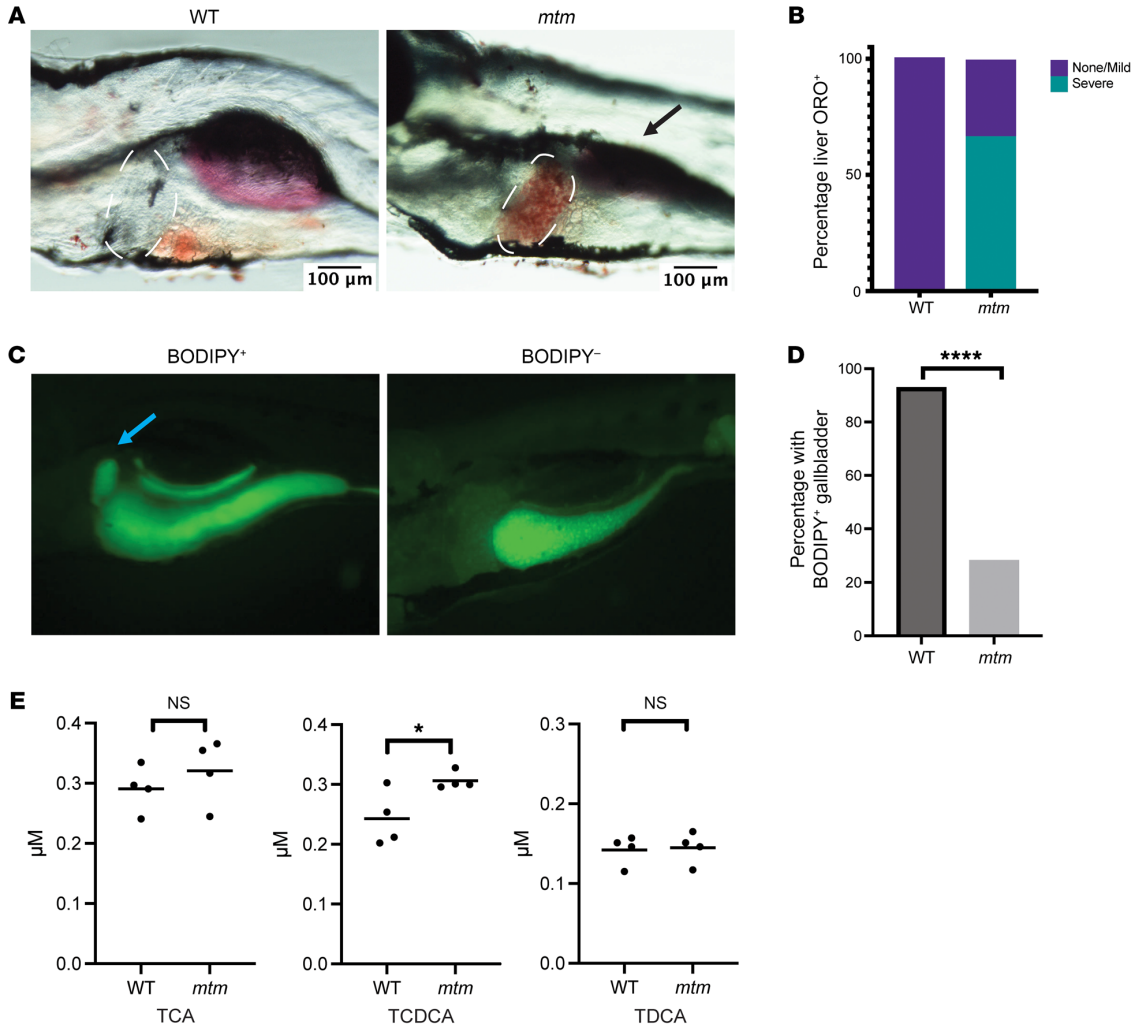
*mtm* zebrafish show evidence of hepatic steatosis and cholestasis. (**A**) Oil Red O staining shows hepatic steatosis in 5 dpf zebrafish larvae. Dashed lines outline the liver, where there is evidence of lipid accumulation in *mtm* zebrafish. Black arrow denotes the swim bladder, which is not properly inflated in *mtm* mutants. Scale bars: 100 μm. (**B**) Quantification of Oil Red O in liver (*n* = 10 fish for each condition). No WT larvae had severe steatosis compared with 66% of *mtm* larvae. (**C**) BODIPY feeding assay showed impaired bile flux in *mtm* larvae. WT and *mtm* zebrafish were fed AP-100 fish food mixed with BODIPY C12. Representative images are shown of fish with positive and negative gallbladder fluorescence. Blue arrow denotes the gallbladder. Original magnification, ×25. (**D**) Quantification of gallbladder fluorescence combining 2 independent biological replicates (*n* = 30 fish per replicate). Ninety-three percent of WT larvae exhibited gallbladder fluorescence after BODIPY exposure, whereas only 28% of *mtm* larvae showed gallbladder fluorescence (****P < 0.0001, by Fisher’s exact test). (**E**) Comparisons of individual bile acids at 5 dpf. TCA, TCDCA, and TDCA are 3 conjugated, hydrophobic bile acids that can cause toxicity when their levels are elevated. TCA and TDCA levels appeared unchanged, whereas TCDCA levels were elevated in *mtm* larvae (*P = 0.039, by unpaired, 2-tailed *t* test).

For this study, we utilized a previously published zebrafish model of X-linked myotubular myopathy (16). This model harbors a loss-of-function mutation in the lone *mtm1* gene (zfin Zf711); biallelic mutation results in the *mtm* phenotype, with progressive fin degeneration, impaired swim behavior, and early death between 7 and 9 days post fertilization (dpf). Homozygous mutants are hereafter referred to as *mtm*. We first verified the previous observation of abnormal lipid accumulation in the liver of this model using Oil Red O, a stain for neutral lipids. We observed substantial staining in the livers of *mtm* larvae at 5 dpf that was completely absent in WT zebrafish (Figure 1, A and B), suggestive of hepatic steatosis. As we were specifically interested in assessing for cholestasis (given the reports in human XLMTM of liver cholestasis), we next examined bile flux using a BODIPY feeding assay, which has previously been used in other fish models of cholestasis (13, 21). We fed 5 dpf larvae powdered fish food mixed with BODIPY-tagged C12. After a 1-hour feeding, we imaged the fish and scored for gallbladder fluorescence, a marker of normal bile flux. On average, 93% of the WT larvae showed BODIPY transit into the gallbladder, whereas in *mtm* larvae the rate was only 28% (Figure 1, C and D). Gut progenitor cell populations that were visualized in *mtm*-knockdown larvae (using a *sox17:GFP* stable transgenic line) appeared similar to those in controls, suggesting that early endoderm development was normal in these fish (Supplemental Figure 1; supplemental material available online with this article; https://doi.org/10.1172/JCI166275DS1). We also quantified the bile salt composition, another potential indication of impaired bile transport, in whole larvae at 5 dpf by liquid chromatography–mass spectrometry (LC-MS). Three bile acids could be detected: taurocholic acid (TCA), taurochenodeoxycholic acid (TCDCA), and taurodeoxycholic acid (TDCA) (Figure 1E). TCA and TDCA levels were unchanged, but we observed elevated levels of TCDCA, a hydrophobic conjugated bile acid associated with hepatic toxicity (22). Overall, these results reveal that *mtm* zebrafish exhibited multiple signs of liver dysfunction consistent with cholestasis.

### mtm zebrafish have alterations in biliary tree organization and structure.

**Figure 2:**
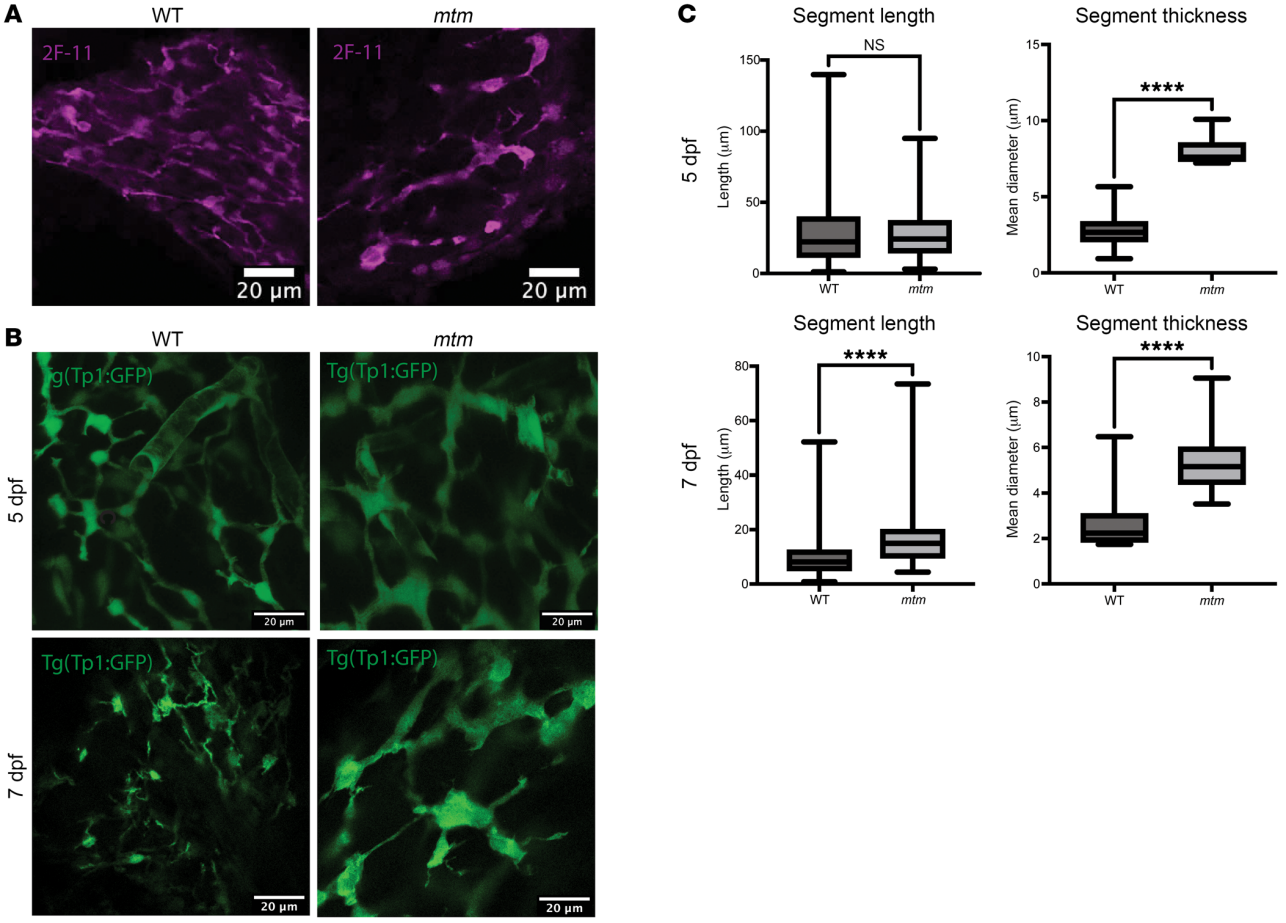
*mtm* zebrafish have dilated bile ducts with reduced branching. (**A**) Whole-mount immunostaining of 7 dpf zebrafish larvae with 2F-11, an antibody that stains bile ducts. The *mtm* biliary tree was simplified and dilated as compared with the WT sibling. Scale bars: 20 μm. (**B**) Live-image analysis of *Tp1:GFP* bile duct transgenic line at 5 dpf and 7 dpf. The simplified and dilated *mtm* bile ducts seen with 2F-11 staining were also observable by this technique. Defects were more severe at 7 dpf compared with 5 dpf. Scale bars: 20 μm. (**C**) Quantification of biliary tree segment length and thickness using the Imaris filaments module. The segment length was unchanged at 5 dpf (WT median = 22.22, *mtm* median = 24.24, *P* = 0.43) but increased in *mtm* larvae by 7 dpf (WT median = 8.14, *mtm* median = 15.0, ****P < 0.0001), suggesting a reduction in branch complexity. Segments were thicker in *mtm* larvae as early as 5 dpf (WT median = 2.27, *mtm* median = 7.60, ****P < 0.0001), and this trend continues at 7 dpf (WT median = 2.24, *mtm* median = 5.15, ****P < 0.0001). Statistical significance was determined by Mann-Whitney *U* test.

We next examined the zebrafish biliary network for structural changes using 2 orthogonal methods: section immunostaining of dissected livers and visualization of transgenic marker zebrafish. Using immunofluorescence staining for 2F-11, a zebrafish-specific antibody that recognizes annexin-a4 and stains the biliary tree (23), we detected widespread disorganization of the biliary system, with reduced branching and areas of aberrant aggregation (Figure 2A). Using live image analysis of a previously published reporter line, *Tg(Tp1:GFP)*, in which *Tp1* is a Notch-responsive element used in zebrafish to label the bile ducts (24), we observed in *mtm* larvae a similar loss of biliary tree complexity and, again, the presence of dilated bile ducts. This phenotype was visible by 5 dpf and worsened by 7 dpf (Figure 2B). Quantification of these images confirmed that both the segment mean diameter and segment length, which can be used to infer the degree of branching, were altered at 7 dpf (Figure 2C). Only the segment mean diameter was increased at 5 dpf, suggesting that dilation of the bile ducts preceded the loss of biliary tree complexity.

### Canalicular transporter localization and expression is altered in mtm zebrafish.

**Figure 3:**
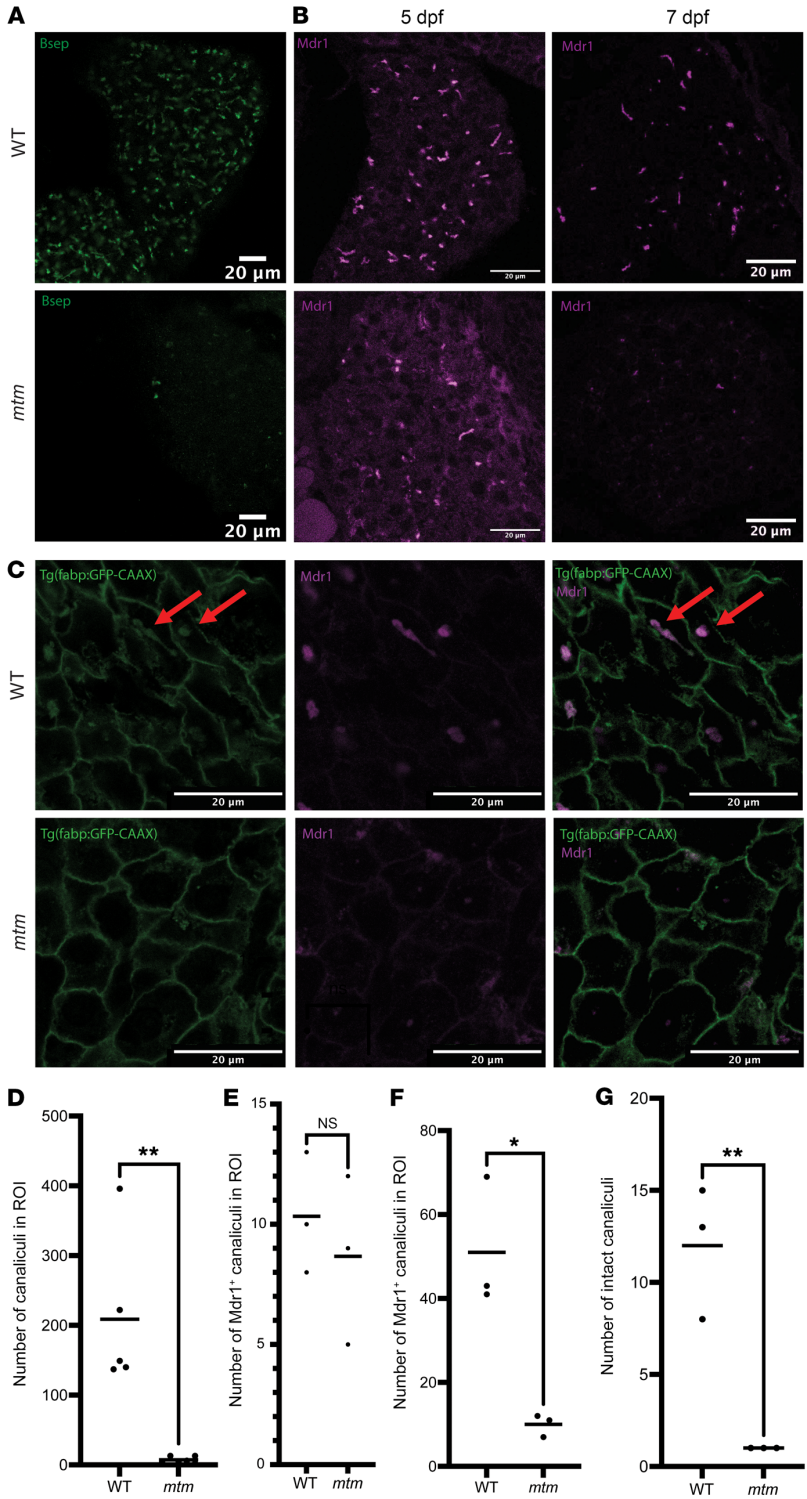
Bile acid transport protein expression is altered in liver from *mtm* zebrafish. (**A**) Immunofluorescence staining of whole-mount zebrafish at 7 dpf using anti-Bsep. In *mtm* larvae, Bsep staining was essentially undetectable. Scale bars: 20 μm. (**B**) Immunofluorescence staining of paraffin sections of whole zebrafish examined at 2 time points: 5 dpf and 7 dpf. Scale bars: 20 μm. (**C**) Coimmunofluorescence staining of sectioned 7 dpf zebrafish for GFP-CAAX, a membrane marker, as well as Mdr1. Costaining revealed reduced canaliculi numbers and altered morphology in *mtm* embryos. As seen in **B**, Mdr1 staining was also absent. Red arrows point to examples of canaliculi that were positive for both GFP-CAAX and Mdr1. Scale bars: 20 μm. (**D**) Quantification of Bsep^+^ canaliculi in WT versus *mtm* larvae in **A** at 7 dpf. Bsep^+^ puncta were reduced in *mtm* larvae (WT mean = 208.8 ± 110.3, *mtm* mean = 7.18 ± 4.71, **P = 0.0014, by unpaired, 2-tailed *t* test). (**E** and **F**) Quantification of Mdr1^+^ canaliculi in sectioned WT and *mtm* larvae from **B**. At 5 dpf (**E**), the WT and *mtm* images had similar numbers of puncta (WT mean = 10.33 ± 2.517, *mtm* mean = 8.667 ± 3.512, *P = 0.5406, by unpaired, 2-tailed *t* test). At 7 dpf, the number of Mdr1^+^ puncta in *mtm* larvae was greatly reduced (WT mean = 51 ± 15.62, *mtm* mean = 10 ± 2.65, **P = 0.011, by unpaired, 2-tailed *t* test). (**G**) Quantification of GFP^+^ canaliculi in **C**. There were significantly more intact canaliculi in WT livers than in *mtm* livers (WT mean = 12.0 ± 3.61, *mtm* mean = 1.0 ± 0, **P = 0.0062, by unpaired, 2-tailed *t* test).

Considering the known cellular role of MTM1 in endosomal sorting, we hypothesized that aberrant trafficking of bile transporters could be the cause of the structural and functional changes we observed in the *mtm* livers (25, 26). Using immunostaining, we examined Bsep and Mdr1, 2 well-known canalicular transporters (Figure 3, A and B) (27). While at 5 dpf Mdr1 protein levels were minimally reduced when compared with WT (Figure 3,B and E), by 7 dpf, both Mdr1 and Bsep protein expression levels were nearly absent in the *mtm* fish, suggesting a nearly complete loss of transporter expression (Figure 3, A, B, D, and F).

On the basis of these observed changes in transporter expression, we sought to assess the structure of the canaliculi in the *mtm* mutants. To do this, we utilized a transgenic line with liver-specific membrane-localized GFP to examine hepatocyte membranes [*Tg*(*fabp10:GFP-CAAX*)]. We first confirmed that we could visualize canaliculi in WT zebrafish by using double staining with anti-MDR1 (Figure 3C). We then looked at *mtm* larvae and found fewer canaliculi as compared with their WT siblings (Figure 3, C and G), corroborating the aberrant canalicular structure suggested by the abnormal Mdr1 and Bsep staining.

**Figure 4:**
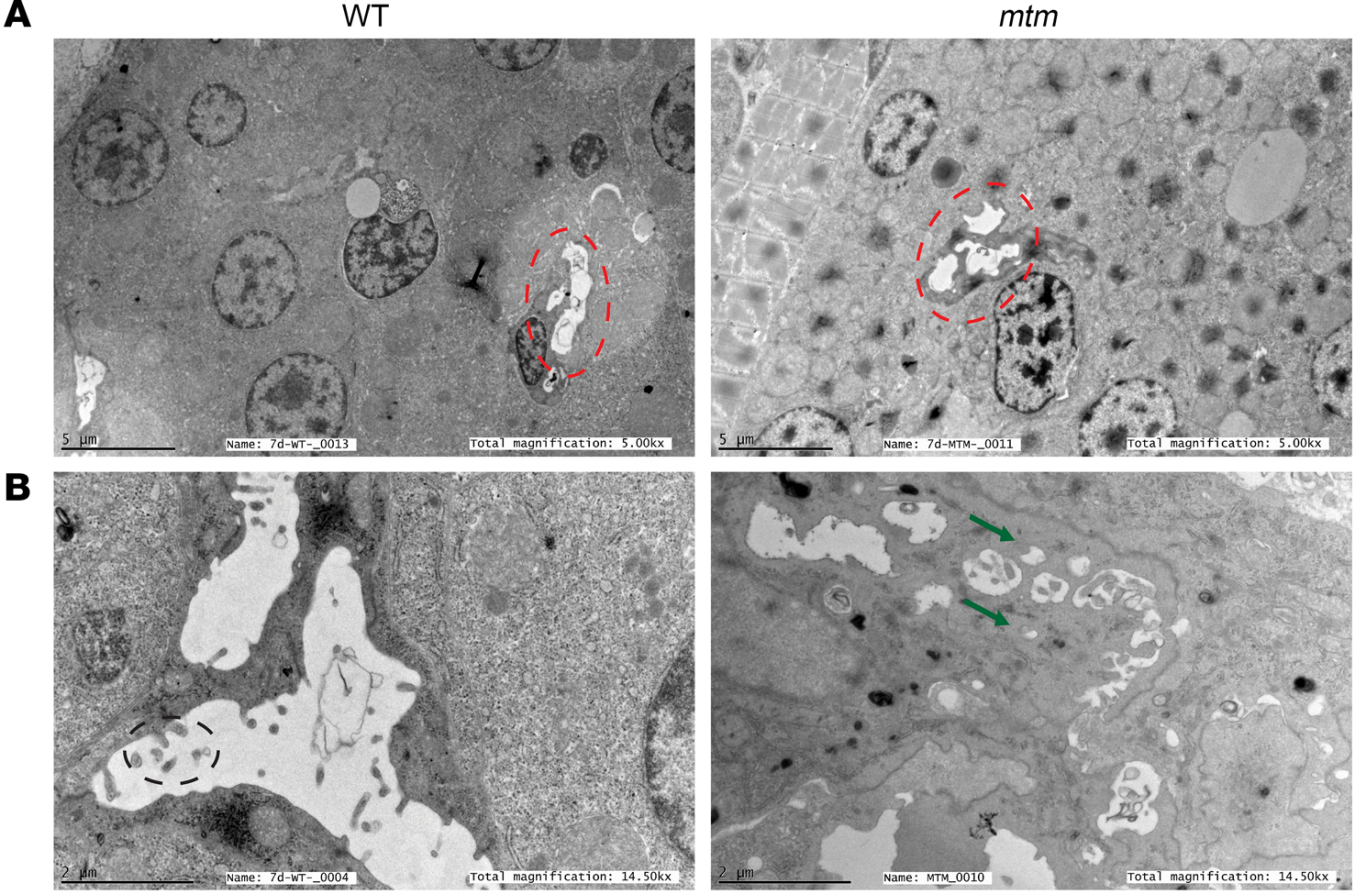
Canalicular ultrastructure is disrupted in *mtm* zebrafish. Electron microscopy of whole 7 dpf zebrafish was used to define liver ultrastructure. (**A**) Magnification (original magnification, ×5,000; scale bars: 5 μm) showing multiple hepatocytes, with bile canaliculi outlined by red dashed lines. Fragmentation of *mtm* canaliculi was already visible at this magnification. (**B**) Higher-magnification visualization of bile canaliculi (original magnification, ×14,000; scale bars: 2 μm). In the WT panel, canalicular microvilli are apparent (black dashed circle). In the *mtm* sample, the canaliculus is devoid of microvilli. Green arrows point to fragmented parts of the canaliculus. *n* = 3 zebrafish per genotype.

To directly visualize the bile canalicular structure, we next performed transmission electron microscopy (TEM) imaging of whole zebrafish larvae. Canaliculi from WT zebrafish exhibited a degree of variability, but all appeared as long, continuous structures (Figure 4A). Also, observable were the canalicular microvilli, which appeared as electron-dense shapes within the canaliculi (Figure 4B). In *mtm* larvae, however, we observed disrupted, fragmented canaliculi, with fewer microvilli were than seen in WT larvae (Figure 4, A and B). The disruption of the canaliculi, in addition to the loss of the transporters, highlights the requirement for Mtm1 in the formation and maintenance of this critical structure.

### mtm1 loss results in a unique transcriptomic signature associated with inflammation but normal bile transporter expression.

**Figure 5:**
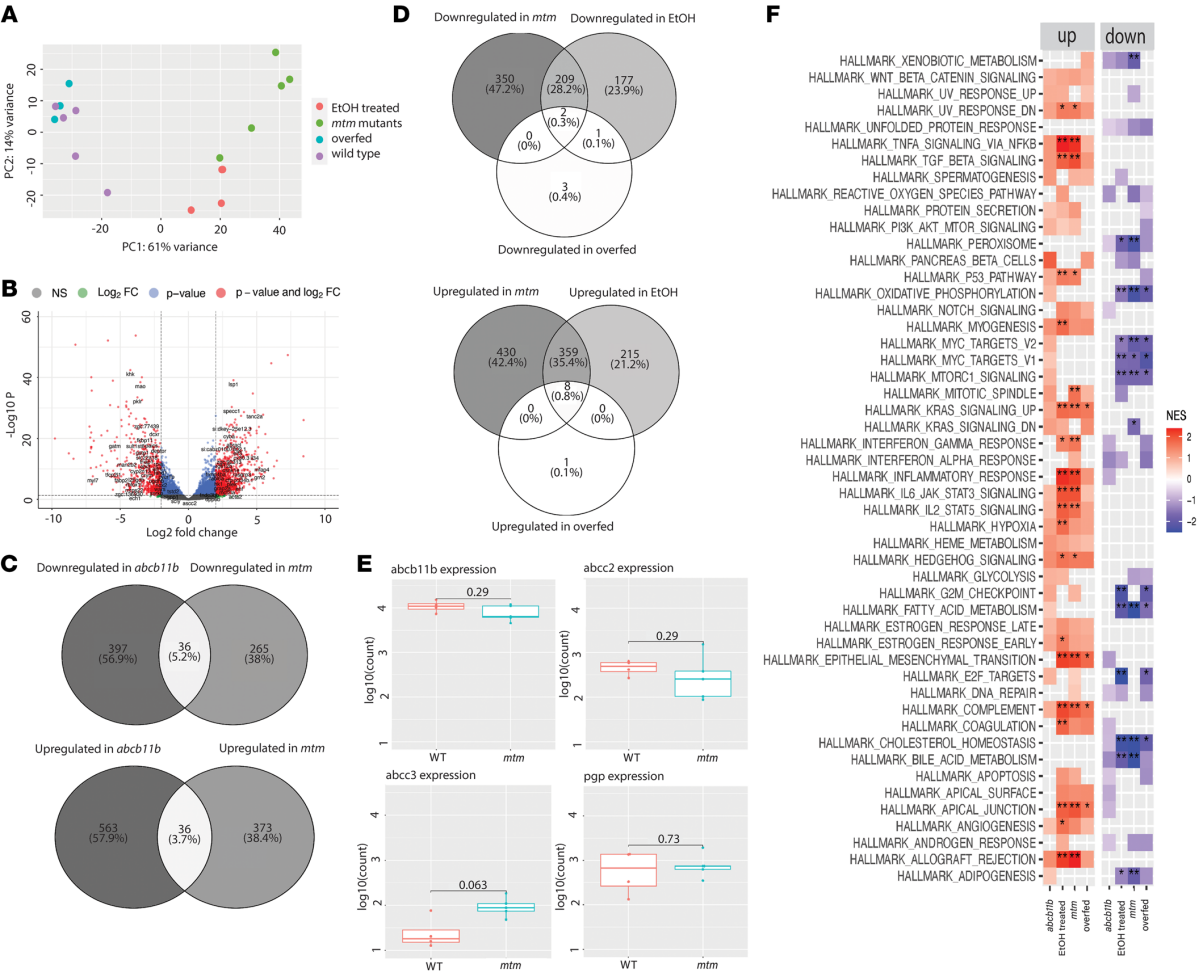
Livers from *mtm* zebrafish exhibit widespread transcriptional changes. Comparative RNA-Seq from isolated livers from 7 dpf larvae from the following conditions: WT, *mtm* mutants, WT exposed to a high-fat diet (overfed), WT exposed to alcohol (EtOH-treated), and *abcb11b* mutants (panel **F** and Supplemental Figure 2). (**A**) PCA shows that transcriptomes from *mtm* zebrafish segregated together and were distinct when compared with WT and the other pathologic conditions. (**B**) Volcano plot showing differential expression between *mtm* and WT livers. There were 797 transcripts upregulated and 561 transcripts downregulated in the *mtm* mutants (absolute [log_2_ fold change (FC)] >2 vs. WT, adjusted *P* = 0.05). (**C**) Venn diagrams comparing *mtm* fish with *abcb11b*-knockout fish. These transcriptomes had little overlap. (**D**) Venn diagrams showing comparisons between *mtm* zebrafish and fish treated with a high-fat diet or alcohol. In general, the individual transcriptomes were distinct and with little overlap. (**E**) Direct interrogation of transcripts from genes encoding canalicular transport proteins. There were no statistically significant changes between WT and *mtm* zebrafish, indicating that the expression changes seen in *bsep* (*abcb11b*) and *mdr1* (*pgp*) were at the posttranscriptional level. (**F**) Pathway analyses of the various conditions using the fgsea (version 1.10.1 R package) analytic tool. The most striking differences were found in pathways representing inflammation and immune responses, which are shared in part between *mtm* and *abcb11b* mutants.

Given the reduced expression of bile transport proteins, we considered whether these alterations might be driven by changes at the transcriptional level. To define transcriptome level differences, we performed comparative bulk RNA-Seq on livers extracted from 5 dpf WT and *mtm* zebrafish, as well as from experimental models of diet-related hepatic steatosis and alcohol-induced liver injuries (28, 29). Overall, we uncovered a unique and aberrant transcriptional profile in *mtm* livers that was distinct from that of WT and both diet- and alcohol related-liver injury as demonstrated by the clustering of each group of samples using principal component analysis (PCA) (Figure 5A). A total of 430 and 350 genes were found to be uniquely upregulated and downregulated, respectively, in *mtm* livers compared with WT (Figure 5, B and D). Overall, the transcriptome profile was highly enriched for genes enriched in inflammatory pathways, consistent with the known involvement of the immune system in responding to cholestatic liver injury (Figure 5F) (30).

In addition to comparing the *mtm* liver transcriptome with models of hepatic steatosis, we also compared these data with RNA-Seq data on livers from *abcb11b*-knockout fish, a genetic zebrafish model of PFIC2 (Figure 5C). Although the 2 transcriptomes mostly exhibited distinct expression changes, there were common changes in both TNF-α signaling and complement pathway genes, suggesting some similarities with respect to inflammation (and similarities with pathways known to be dysregulated in cholestasis, as mentioned above).

Importantly, we interrogated the RNA expression of known canalicular transporters including *mdr1* and *abcb11b* and found no statistically significant differences between WT and *mtm* livers, suggesting that the changes seen in transport expression were occurring at the protein level (Figure 5E and Supplemental Figure 2C). Finally, we also compared the *mtm* liver transcriptome with that of isolated tails of *mtm* knockouts, which were enriched in muscle tissue (Supplemental Figure 2B). There were very few shared differentially regulated genes between the 2 samples and nearly no overlap with respect to pathway enrichment. This suggests that the changes identified in either sample represented tissue-specific changes and not global defects seen across cell types secondary to the loss of *mtm1*.

### Rab11 localization and expression is altered in mtm zebrafish.

**Figure 6:**
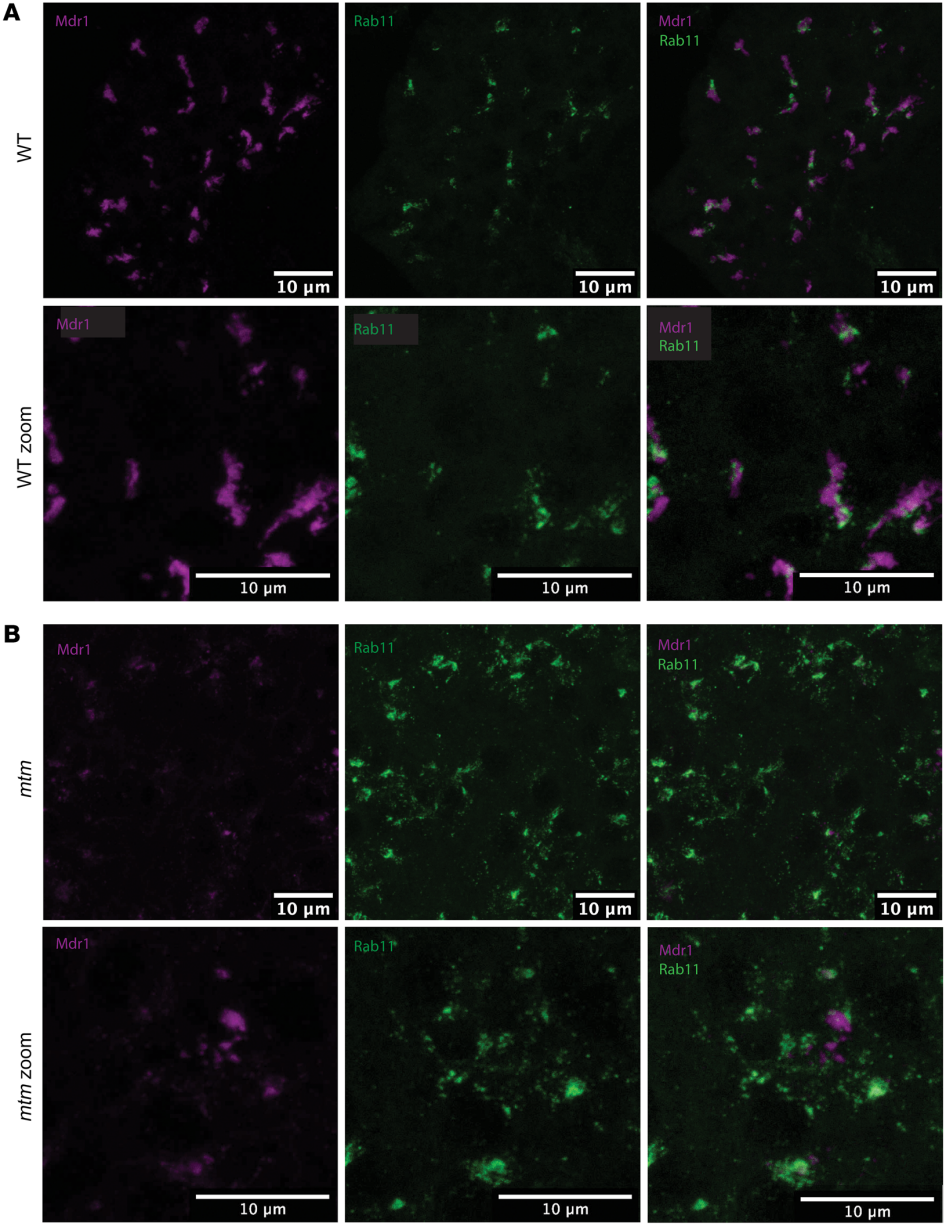
Altered recycling endosomal trafficking in *mtm* livers. Confocal images of 7 dpf zebrafish sections immunostained for Mdr1 (purple) and Rab11 (green), shown at lower and higher magnification. Mdr1 is a canalicular transporter, and Rab11 is a GTPase found on recycling endosomes. (**A**) Rab11 clustered around the canaliculi in WT larvae. (**B**) In *mtm* larvae, Rab11 localization was more diffuse throughout the cytoplasm. Scale bars: 10 μm (zoom view of the images above, ×2.3).

Since bile transporter gene products are not reduced at the transcriptional level, we sought other explanations for the reduced gene transporter expression at the bile canaliculi. Bile transporter localization to the canalicular membrane is mediated by trafficking through PI(3)P/Rab11-positive subapical compartments (SACs) (31). We therefore examined Rab11 distribution in WT and *mtm* larvae. In WT larvae, Rab11 clustered around the canaliculi, in close association with Mdr1 (Figure 6A). In contrast, in *mtm* larvae, Rab11 was found to be dispersed throughout the cytoplasm, in large cytoplasmic aggregates with loss of pericanalicular localization (Figure 6B). This suggests that the Rab11 trafficking complex was disrupted in *mtm* zebrafish and presents a possible mechanism by which bile transporter gene expression was reduced (i.e., failure to properly traffic to the canalicular membrane).

### Mtm1-GFP localizes to the bile canaliculus and plasma membrane.

**Figure 7:**
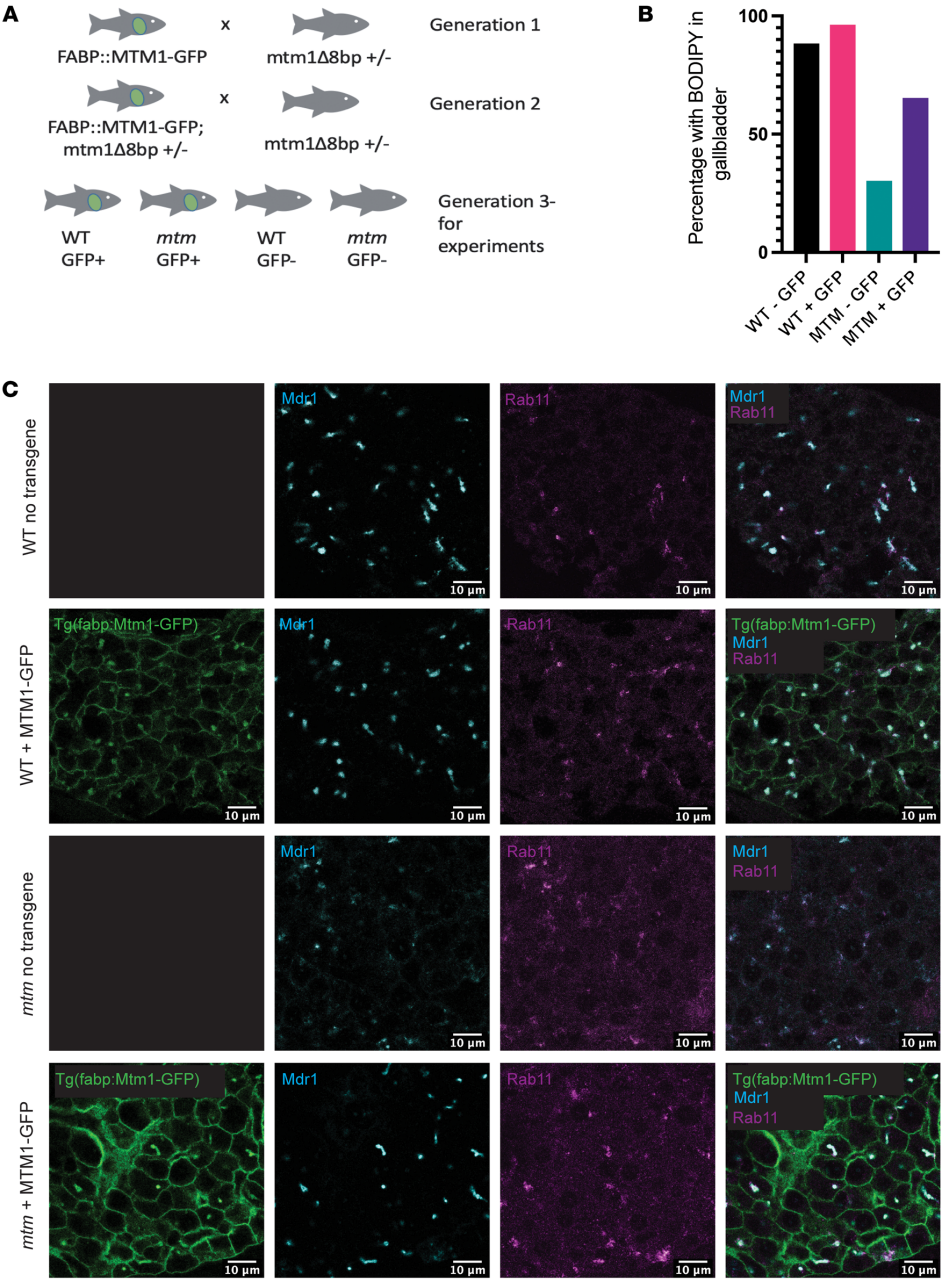
Liver-specific Mtm1 expression rescues the cholestatic phenotype of *mtm* zebrafish. The *fabp:mtm1-GFP* transgene was introduced into the *mtm*-mutant zebrafish line. The resulting fish were analyzed for morphological and functional changes associated with cholestasis. (**A**) Crossing scheme for introducing the *fabp:mtm1-gfp* transgene into the *mtm* zebrafish line. Transgenic fish were outcrossed twice to *mtm1+/*Δ*8* fish, resulting in clutches of larvae for experiments that contained WT and *mtm* fish with and without the transgene. (**B**) A BODIPY assay was used to measure bile flux (WT – GFP = 88%, WT + GFP = 96%, mtm – GFP = 30%, mtm + GFP = 65%). In the *mtm* transgene–positive group, there were more *mtm* mutants with normal bile flux when compared with the *mtm* transgene–negative group, as measured by positive gall bladder fluorescence (*P* = 0.0532, 1-sided Fisher’s exact test). (**C**) Visualization of liver-specific Mtm1 expression from the *fabp:mtm1-GFP* transgene. In WT fish, Mtm1 localized to the plasma membrane and to subapical structures that were Mdr1^+^ and Rab11^+^ by immunostaining (top two rows). In *mtm* zebrafish, hepatocyte-expressed Mtm1 restored bile canalicular architecture and bile transporter expression to the bile canaliculi. Coimmunostaining of whole-mount embryos revealed the reexpression of Mdr1 puncta in 7 dpf *mtm* larvae. Scale bars: 10 μm.

In order to visualize the localization of Mtm1 in the liver, we generated a *Tg*(*fabp:mtm1-GFP)* transgenic line that drives expression of Mtm1 tagged with GFP in all hepatocytes (Figure 7A). Examination of its subcellular localization in WT fish revealed expression at the plasma membrane and bile canaliculi. Using coimmunostaining, we determined that the canalicular fraction of Mtm1-GFP colocalized with Mdr1 and Rab11 (Figure 7C).

### Hepatocyte reexpression of Mtm1 rescues the cholestatic phenotype of mtm zebrafish.

We next used the *mtm1-GFP* transgene to restore *mtm1* expression exclusively to hepatocytes by crossing the transgenic line into our *mtm* zebrafish. Upon reexpression in the *mtm* mutants, we found that expression of both Mdr1 and Rab11 proteins was increased and that they were restored to their normal localization at the canaliculus (Figure 7C). Furthermore, we observed functional improvement of bile transport, as the abnormal BODIPY staining seen in *mtm* larvae was ameliorated, albeit not to WT levels (Figure 7B). Hepatocyte reexpression of Mtm1 thus appeared to be sufficient to rescue (at least in part) the cholestatic defects seen in *mtm* zebrafish. Of note, we still observed increased Oil Red O staining, as well as dilation of the biliary tree, suggesting that the steatosis and dilated bile duct phenotypes may be driven by Mtm1 expression and function in cell types other than hepatocytes (Supplemental Figure 3).

### Targeted chemical screen identifies a DNM2 inhibitor as a modifier of mtm liver phenotypes.

**Figure 8:**
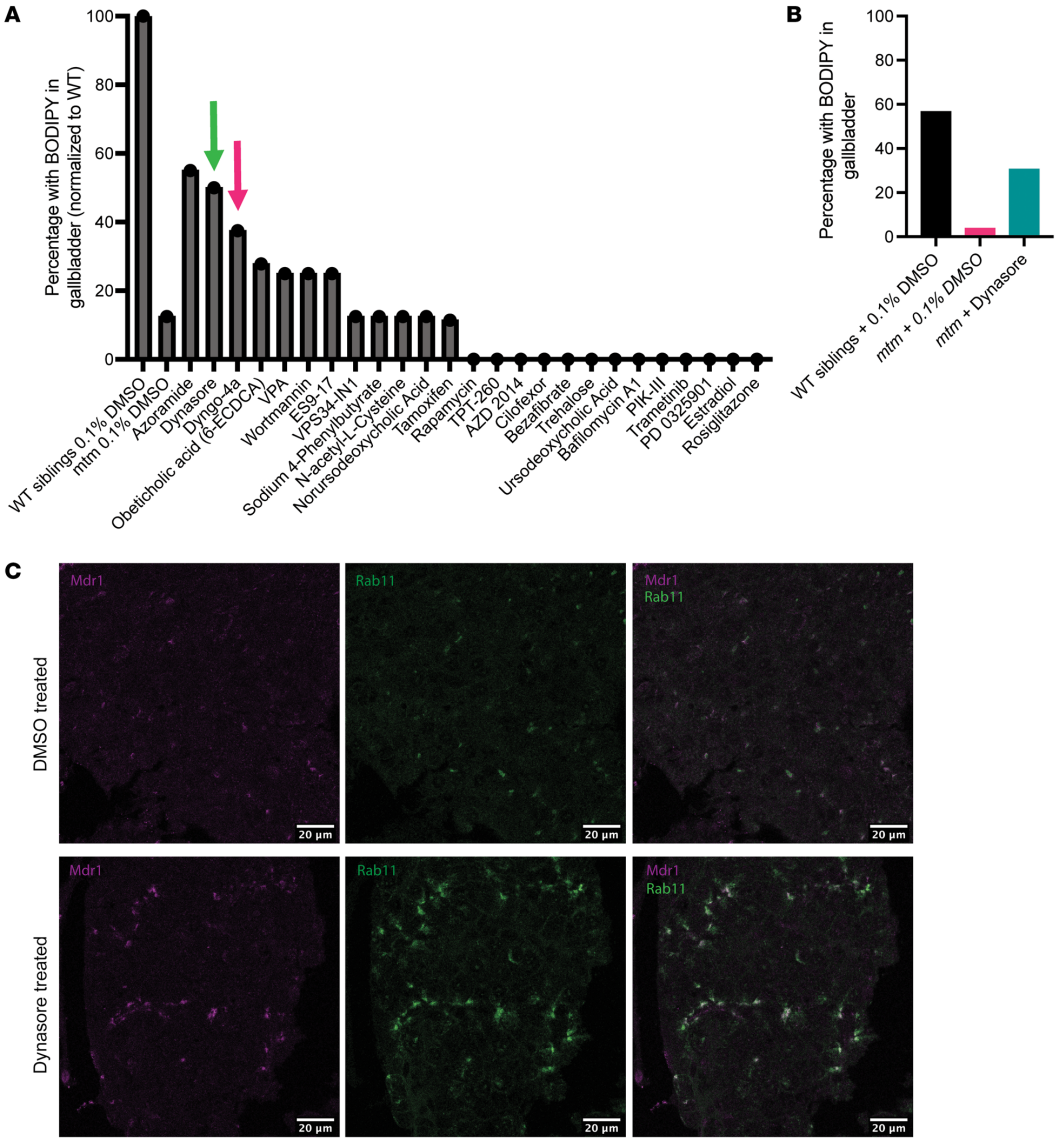
DNM2 inhibition rescues the *mtm* cholestatic phenotype. A targeted panel of chemicals was tested in *mtm* zebrafish, using the BODIPY assay as a screen for the cholestatic liver phenotype. For each chemical, the percentage of *mtm* zebrafish with positive staining in the gallbladder was measured as the readout (*n* = 10 per trial, with 1 replicate per trial). (**A**) Two DNM2 inhibitors, Dynasore (green arrow) and Dyngo-4a (pink arrow), were among the chemicals that produced the highest percentage of BODIPY^+^ larvae. (**B**) Validation of Dynasore combining 3 independent replicates of 10 larvae per replicate. Dynasore-treated *mtm* larvae had improved bile flux compared with their DMSO-treated *mtm* siblings (Dynasore = 30.6%, DMSO = 3.67%, *P* = 0.019, by 2-sided Fisher’s exact test) and did not differ significantly from their WT DMSO-treated siblings (WT percentage = 56.7%, Dynasore percentage = 30.6%, *P* = 0.13, 2-sided Fisher’s exact test). (**C**) Dynasore partially restored canalicular structure and transporter expression. Coimmunostaining was performed on whole-mount embryos either exposed to DMSO or treated with Dynasore. As expected, in DMSO-treated *mtm* zebrafish, essentially no Mdr1 staining was appreciated. In Dynasore-treated larvae, however, there was robust reexpression of Mdr1 and proper colocalization with Rab11. Scale bars: 20 μm.

Using our previous knowledge of modifiers of the muscle phenotype of XLMTM (16, 25, 32–36), we performed a small-scale chemical modifier screen using the BODIPY assay as a readout (Figure 8A). *mtm* zebrafish were treated with chemicals at 3 dpf onward and later fed twice for 1 hour with BODIPY — once at 5 dpf and once at 6 dpf. Fish were screened for gallbladder fluorescence after the 6 dpf feeding. One class of chemicals, DNM2 inhibitors, produced 2 positive hits on our screen: Dynasore, a pan-dynamin inhibitor that also influences cholesterol homeostasis (37), and Dyngo-4a, a DNM2-specific inhibitor (38). Both drugs partially rescued the bile flow phenotype across 3 replicates (Figure 8B). We then sought (for Dynasore) to determine whether this functional rescue is associated with improvement in canalicular structure. We did this by immunostaining for Mdr1 and Rab11 and found that localization of both proteins was restored to their WT locations, albeit with qualitatively lower protein levels (Figure 8C).

## Discussion

The emergence of fatal liver disease as an adverse event in the gene therapy trial for X-linked myotubular myopathy and the finding of intrahepatic cholestasis in untreated patients (8) have raised the question of whether loss of MTM1 itself is associated with liver pathology and, if so, via what mechanism. In this study, we conclusively demonstrate, using the zebrafish model system, that *mtm1* mutation can directly result in liver abnormalities with cholestatic features. Furthermore, these liver changes were hepatocyte autonomous, as they could be rescued by hepatocyte-exclusive reexpression of the *mtm1* gene and were likely related to aberrant membrane trafficking. Finally, we show in a pilot chemical screen that DNM2 inhibition could ameliorate aspects of the liver phenotype, thus presenting a potential pathway for treating this emerging and serious aspect of the disease.

MTM1 is a ubiquitously expressed protein with a critical function in vitro in regulating vesicular sorting through the endosome (39). Its function in vivo has remained much less well established, despite the plethora of knowledge related to the consequences of its loss in skeletal muscle. Furthermore, a requirement for MTM1 in nonmuscle tissues has yet to be established. In this study, we provide convincing evidence of a role for MTM1 in the liver and present data to suggest a potential function. Specifically, using a transgenic Mtm1-GFP construct, we visualized Mtm1 in the liver at the plasma membrane and in close approximation to a Rab11 compartment that contains bile acid transport proteins. This compartment is likely the subapical compartment that is enriched with PI(3)P, one of MTM1’s lipid substrates (40, 41). Rab11 is implicated in the transport of bile transport proteins to the canaliculus and probably does so in a PI(3)P-dependent fashion. We thus hypothesized that MTM1’s function in the liver is to control (via Rab11 regulation) transporter trafficking to the canalicular membrane (Supplemental Figure 4). Interestingly, pathogenic variants in several components of the endosomal system (e.g., in VP16 and VPS33A) cause cholestatic liver disease in humans that is accurately modeled by loss-of-function mutations in the zebrafish (42, 43). Thus, overall, it appears very possible that the endosomal system is critically at the intersection of canalicular membrane formation and maintenance and is required for normal bile acid transport to occur.

The pathology identified in *mtm* zebrafish was consistent with both cholestasis and hepatic steatosis, abnormalities in keeping with the those thus far reported in patients with XLMTM (8). The loss of BSEP protein expression in the *mtm* zebrafish was intriguing because, in humans, mutations in *ABCB11* (the gene that encodes BSEP) are associated with 2 forms of cholestatic liver disease: progressive familial intrahepatic cholestasis (PFIC) and benign recurrent intrahepatic cholestasis (BRIC) (12). The fact that recurrent episodes of pauci-symptomatic cholestasis have been described in a few patients with XLMTM suggests shared symptomatology with BRIC. If, like the *mtm* zebrafish, patients with XLMTM also demonstrate reduced BSEP protein expression, the 2 disorders may in fact be similar in many ways (8), and/or variability in underlying *ABCB11* gene expression may be a critical modifier of the XLMTM liver phenotype. Of note, decreased BSEP expression was observed in liver biopsy samples from patients with XLMTM in a recent case series (8). These patients were excluded for pathogenic variants in *ABCB11* (8), although nonpathogenic expression–modifying variants (e.g., expression quantitative trait loci [eQTLs]) were not evaluated.

In terms of the severity and/or frequency of events in patients, we speculate that they may be triggered by environmental factors such as diet and/or infection. All 5 patients in the recent case series had suspected antecedent viral infection. Such potential precipitants will be explored in future experiments using our zebrafish model. The interrelationship with progressive, fatal cholestatic liver disease in XLMTM and adeno-associated virus 8 (AAV8) exposure also remains to be better understood. Unfortunately, zebrafish do not readily take up AAV (44), and thus at present we are not able to evaluate this interaction. Therefore, there is a need to model XLMTM-related liver disease in other systems, or else to modify the zebrafish in some way so that it can be infected with AAV. In this regard, induced pluripotent stem cell–derived (iPSC-derived) models are a promising consideration, and experimentation is currently underway in our laboratory to establish hepatocytes from iPSCs with *MTM1* mutations (45).

Obviously, an important goal of this work is to identify potential interventions that may ameliorate or prevent episodes of cholestasis in patients, particularly in the setting of AAV exposure. To address this, we performed a pilot chemical screen and identified two DNM2 inhibitors as chemicals that can improve bile flux. While these specific inhibitors have not been used in humans, a genetic strategy for lowering DNM2 expression has recently been tested in a limited clinical trial for XLMTM. This strategy uses an antisense oligonucleotide to reduce DNM2 mRNA and protein levels (34). It showed great success at improving the skeletal muscle pathology in the mouse model of XLMTM but met with dose-limiting toxicity in the recent first-in-human clinical trial. One consideration may be that a short course of DNM2 inhibition (either genetic or chemical), given around the time of gene therapy, could reduce liver toxicity and enable successful transduction into the skeletal muscle. Given DNM2’s well-recognized role as a regulator of endocytosis and membrane trafficking, the fact that DNM2 inhibition improved the liver phenotype in our *mtm* zebrafish reinforces our hypotheses that MTM1 functions as a regulator of bile transporter trafficking and that MTM1-dependent abnormalities in the canalicular membrane are due to defective endosomal function.

In total, using the zebrafish model system, we show that Mtm1 is required for the normal formation and maintenance of liver structure and function and specifically identify a role for Mtm1 in canalicular structure and function. We uncovered a potential therapeutic strategy for MTM1-related liver disease (i.e., DNM2 inhibition) and set the groundwork for future investigation into MTM1 liver function and into factors and triggers that may modulate the cholestatic phenotype.

## Methods

### Zebrafish maintenance.

Zebrafish (AB strain) were raised at 28.5°C and maintained at the Zebrafish Facility at the Hospital for Sick Children in Toronto, Ontario, Canada. Experiments were performed on embryos and larvae between the ages of 3 and 7 days.

### Oil Red O staining.

At 5 dpf, zebrafish were fixed overnight in 4% PFA and washed 3 times in PBS-T (PBS plus 0.1% Tween). Fish were stained for 15 minutes in a mixture of 300 μL Oil Red O mixture (0.5% Oil Red O in 100% isopropyl alcohol) and 200 μL double-distilled H2O (ddH_2_O). After staining, fish were rinsed 3 times with PBS-T and then twice more in 60% isopropyl alcohol diluted in water. Fish were then mounted in glycerol and imaged.

### BODIPY feeding experiments.

BODIPY C12 was reconstituted in 1 mL 100% ethanol and mixed with 1 mL chloroform. This was then combined with 0.5 g AP-100 and left to evaporate for 3 hours in a fume hood. Thirty 5 dpf zebrafish from each condition were fed 4.5 mg AP-100/BODIPY for 1 hour and then anesthetized and imaged on a Zeiss Axio Zoom macroscope. Fish were scored for fluorescence in the intestine (indicating that they had fed) and then for fluorescence in the gallbladder.

### Bile acid quantification.

Zebrafish (5 dpf) were anesthetized in 0.1% tricaine and frozen at –80°C. They were then shipped to the Baylor Research Institute Center for Metabolomics (Houston, Texas, USA), where bile acids were quantified using the AbsoluteIDQ Bile Acids Kit (Biocrates).

### Whole-mount immunofluorescence staining.

Zebrafish (57 dpf) were anesthetized in 0.1% tricaine and fixed in 4% PFA overnight at 4°C. The next day, they were washed 3 times in PBSTx (PBS plus 1% Triton X-100), permeabilized in acetone for 1 hour at –80°C, washed twice in ddH_2_O and PBSTx, blocked for 2 hours in 5% BSA plus 10% goat serum, and incubated with primary antibodies overnight at 4°C. The next morning, fish were washed 8 times for 15 minutes each in PBSTx and incubated overnight with a secondary antibody at 4°C. On the third day, the samples were washed 8 times for 15 minutes each with PBSTx, bleached using a mixture of H_2_O_2_ and KOH for 10 minutes, washed 3 times more in PBSTx, and then imaged on a Leica SP8 Lightning confocal microscope.

### Immunofluorescence staining of zebrafish sections.

At 5–7 dpf, zebrafish were anesthetized in 0.1% tricaine and fixed in 10% formalin overnight at room temperature (RT), and then embedded in paraffin blocks and sectioned at 2–4 nm thickness. Slides were dewaxed through 2 changes of xylene and hydrated through 100%, 90%, and 70% ethanol. Antigen retrieval was performed using a pressure cooker, and then slides were treated with H_2_O_2_ and methanol for peroxidase inhibition. Blocking was performed using 10% goat serum in PBS plus 0.1% Tween 20 (PBS-T) for 1 hour at RT, and then slides were incubated with a primary antibody for 1 hour, washed for 15 minutes in PBS-T, and incubated 30 minutes in secondary antibody. Slides were mounted using Prolong Diamond and cured at RT for 48 hours before proceeding to imaging on a Leica SP8 Lightning confocal microscope. The following antibodies were used: Mdr1 (Thermo Fisher Scientific, BS-0563R, 1:100); BSEP (MilliporeSigma, HPA019035, 1:100); BSEP (Abcam, ab155421, 1:200); GFP (Abcam, ab252881, 1:100); Rab11 (Genetex, GTX127328, 1:300); and 2F-11 (Abcam, ab71286, 1:100).

### Transgenic zebrafish lines.

The *Tg*(*Tp1:GFP)* line (ZDB-ALT-090625-1) and the *Tg(fabp10:GFP-CAAX*) line (ZDB-ALT-160405-2) were both imported from the Zebrafish International Resource Center (ZIRC).

### Bile canaliculi and biliary tree quantification.

Bile canaliculi quantification was performed using the Imaris 10 spots module. Five *Z*-stacks each of WT and *mtm* livers were input, using the same size region of interest (ROI) for each image. Bile duct quantification was performed using the Imaris 10 filaments module. Entire *Z*-stacks were imported into the software, and the filaments wizard was used to define soma and segments. Segment length and mean segment diameter were used for statistical analysis.

### TEM.

Zebrafish (5–7 dpf) were anesthetized in 0.1% tricaine and fixed in Karnovsky’s fixative (2.5% glutaraldehyde plus 2% PFA in 0.1 M cacodylate buffer, pH 7.5) overnight at 4°C. Samples were then washed 3 times for 5 minutes each in 0.1 M cacodylate buffer, postfixed in 1% osmium for 1.5 hours at RT, and then washed again. Samples were dehydrated through serial ethanol washes (70%, 90%, 95%, 100%), infiltrated with Epon, and embedded in Epon in a 60°C oven for 24–48 hours. A Leica Ultracut ultramicrotome was used to cut semi-thin (1 μm) and ultra-thin (90 nm) sections, which were then transferred to 200 nm copper grids. Grids were stained with 2% uranyl acetate at RT for 20 minutes, washed 7 times with MilliQ water, stained with lead citrate for 5 minutes, and then washed 7 times again. Samples were imaged using an FEI Tecnai 20 transmission electron microscope.

### Mtm1 rescue.

*The Tg*(*fabp:mtm1-GFP*) line was generated by gateway cloning. A middle entry clone possessing the zebrafish *mtm1* full-length coding sequence was purchased from Thermo Fisher Scientific. *Tg*(*fabp:mtm1-GFP*) zebrafish were crossed into the *mtm1+/*Δ*8* background and raised to adulthood. Offspring were again crossed into the *mtm1+/*Δ*8* background for experiments.

### BODIPY drug screen.

At 3 dpf, 10 *mtm* zebrafish were placed into 24-well plates, each containing 2 mL system water and a chemical (in 0.1% DMSO vehicle). At 5 dpf, the fish were fed once with AP-100 mixed with BODIPY. At 6 dpf, fish were fed again with AP-100/BODIPY and screened for gut and gallbladder fluorescence. Compounds screened and concentrations can be found in Supplemental Table 1.

### Comparative transcriptomic experiment and analysis.

Forty livers per replicate (*n* = 4–5 replicates per group) were dissected under a bright-field microscope at 7 dpf and then pooled prior to RNA extraction using the QIAGEN RNAeasy Micro kit. Animals in the overfed group were given 6 times the normal amount of food at each feeding from day 5 to day 7, while EtOH-treated animals were exposed to 2% ethanol for 32 hours prior to sample collection. The presence of hepatic steatosis was confirmed in both groups.

Samples were submitted to The Centre for Applied Genomics (TCAG). The SMART-Seq v4 Ultra Low Input RNA kit (Takara Bio) was used for the cDNA conversion. Library preparation followed the protocol of the Nextera XT library prep kit. Libraries were quality controlled (run on the Bioanalyzer DNA High Sensitivity chip to check for size and quantified by quantitative PCR (qPCR) using the Kapa Library Quantification Illumina/ABI Prism Kit protocol). All libraries were pooled and sequenced on 2 lanes on the Illumina NovaSeq SP flowcell paired-end 2 × 100 bp.

Quality control (QC) and analysis of the RNA-Seq data were performed with the Audentes internally developed bioinformatic pipeline. AWS cloud computing platform was used for the analyses.

FASTQ files from the different runs were merged using “cat.” The merged paired FASTQ files for each sample were run through FastQC (version 0.11.09) to obtain general QC metrics. Adapters were trimmed by Trim Galore (version 0.6.6). Salmon3 (version 1.4.0) was used for our alignment-free pipeline. Adapter-trimmed reads were used as input. The genome index built from the *Danio rerio* reference genome (GRCz11) with associated transcript annotations (GENCODE, version GRCz11.103) was used.

We quantified gene expression using raw counts and kept the genes that showed at least 5 reads in 80% of the samples. We performed differential expression gene testing with DESeq2 (version 1.24.0, R package) using default settings. Replicate was used as covariates within the DESeq2 model. Statistical significance was set a 5% FDR (Benjamini-Hochberg). The Bioconductor package fgsea (version 1.10.1, R package), was used for gene set enrichment analysis (GSEA). Differential expression analysis was conducted for mtm-mutant livers versus WT as well as for 2 other models of liver steatosis (ethanol-treated embryos and overfed embryos) against the WT.

### RNA-Seq for liver samples from WT and abcb11b mutants.

Total mRNAs were extracted from pools of dissected livers from 6 dpf *abcb11b* mutants and WT siblings, 20 livers per replicate and 3 replicates per genotype, using an Arcturus PicoPure RNA isolation kit (Applied Biosystems, KIT0204). Samples were submitted to Novogene for library preparation and RNA-Seq using the vendor’s standard protocol. Bioinformatics analysis was performed as described above.

### qPCR.

A total of 25 whole 5 dpf zebrafish larvae were collected for each condition, and RNA was extracted using the RNeasy Mini kit (QIAGEN, 74106). Three biological replicates were used for each condition. cDNA was made using the High Capacity RNA to CDNA kit (Thermo Fisher Scientific, 4387406), and qPCR was performed using the StepOnePlus qPCR system (Thermo Fisher Scientific, 4376600). The primers used are listed in Supplemental Table 2.

### Zebrafish morpholino injections.

*Tg*(*sox17:GFP*) embryos were injected at the 1-cell stage with 3 nL of 0.6 mM Mtm1 morpholino (ZDB-MRPHLNO-100325-1) (1). They were then examined at 2 dpf on a Nikon A1R confocal. Fish were later checked for fin fold degradation to confirm Mtm1 knockdown.

### Statistics.

All statistical analyses were performed using GraphPad Prism (GraphPad Software), and a *P* value of less than 0.05 was used to determine statistical significance. BODIPY gallbladder fluorescence (Figure 1D) was analyzed with a 2-tailed Fisher’s exact test. Bile acid quantifications (Figure 1E) were analyzed with an unpaired, 2-tailed *t* test. Bile duct complexity data (Figure 2C) were analyzed with a 2-tailed Mann-Whitney *U* test. Quantification of bile canaliculi (Figure 2, D–G) was analyzed with an unpaired, 2-tailed *t* test. Rescue of the BODIPY phenotype (Figure 7B) was analyzed with a 1-tailed Fisher’s exact test. The BODIPY results after Dynasore treatment (Figure 8B) were analyzed with a 2-tailed Fisher’s exact test. Segment length and mean segment diameter were used for statistical analysis by Mann Whitney *U* test in GraphPad Prism (GraphPad Software).

### Study approval.

All zebrafish protocols used in this study were approved by the Animal Care Committee at the Peter Gilgan Centre for Research and Learning (PGCRL) (Toronto, Ontario, Canada) at the Hospital for Sick Children (protocol 1000052731), with the exception of the *abcb11b* transcriptomic data studies, which were performed under the approval of the Cincinnati Children’s Hospital Medical Center IACUC (protocol A3108-01). All zebrafish procedures were performed in strict accordance with the Animals for Research Act of Ontario and the Guidelines of the Canadian Council on Animal Care.

### Data availability.

All RNA-Seq data have been deposited in the NCBI’s Gene Expression Omnibus (GEO) database (GEO GSE235571). Raw data supporting all graphs can be found in the Supporting Data Values file.

## Author contributions

SK, ARD, and JJD conceptualized the study. Data acquisition and interpretation: SK, ARD, EA, GA, JV, MWL, JLE, CY, and JJD acquired and interpreted data. ARD and JJD supervised the study. SK wrote the original draft of the manuscript. SK, ARD, EA, BMK, GA, MWL, JV, CY, and JJD reviewed and edited the manuscript.

## Supplementary Material

Supplemental data

Supporting data values

## Version 1 07/25/2023

In-Press Preview

## Version 2 09/15/2023

Electronic publication
