# Supplementary material for: Loss of Mtm1 causes cholestatic liver disease in a model of X-linked myotubular myopathy
Source: J Clin Invest. 2023 Sep 15;133(18):e166275. doi: 10.1172/JCI166275 (PMC10503795; doi:10.1172/JCI166275)
Supplement: Supplemental data [file jci-133-166275-s209.pdf]

Supplemental Data:

Supplemental Table 1. Chemicals used in Zebrafish BODIPY Screen

Chemical Name	Concentration	Ordering Information	Proportion rescued in Fig 8A
Trehalose	50mM		0
Valproic acid (VPA)	250µM	Sigma, P4543	0.250
Dyngo-4a	12.5µM	Cedarlane, S7163	0.375
Dynasore	25µM	Cedarlane, S8047	0.500
Wortmannin	0.1µM	Cedarlane, S2758	0.250
Trametinib	100nM	Cedarlane, 16292-25	0
AZD 2014	1µM	Cedarlane, 17378-5	0
VPS34-IN1	1µM	Cedarlane, 17392-5	0.125
Sodium 4-phenylbutyrate	500µM	Cedarlane, 11323-1	0.125
N-Acetyl-L-Cysteine	200µM	Cedarlane, 20261-10	0.125
Obeticholic acid	5µM	Cedarlane, 11031-5	0.2778
Cilofexor	1µM	Cedarlane, 25747-1	0
Bezafibrate	100µM	Cedarlane, 10009145-1	0
Ursodeoxycholic acid	100µM	Cedarlane, 15121-250	0
Norursodeoxycholic acid	10µM	Cedarlane, 33925-1	0.125
Bafilomycin A1	0.002µM	Cedarlane, 11038-500	0
Rapamycin	1µM	Cedarlane, 13346-5	0
PIK-III	1µM	Cedarlane, 17002-1	0
Azoramide	10µM	Cedarlane, 18045-10	0.556
PD 0325901	100nM	Cedarlane, 13034-5	0
TPT-260	2.5µM	Cedarlane, 16079	0
ES9-17	10µM	Chembridge, 7577817	0.250
Estradiol	10µM	Sigma, E2758-1G	0
Rosiglitazone	10µM	Cedarlane, 71740	0
Tamoxifen	2uM	Sigma, H7904	0.114

Supplemental Table 2. Primers used in qPCR

Gene	Primers
abcb11b	F- ATTTCCGCAGCAAAGAAGGC<br>R- GTTTTTGACCCCGGGAAAGC
abcc2	F- ACTGCGGTTGTTGTACCGAT<br>R- GCCGGATACGGTTTCTCCAA
ppib	F- CTCAGTTCTTCATCACCACAGTCC<br>R- AGAGGTTTATCTCTCCCGTCCGTC

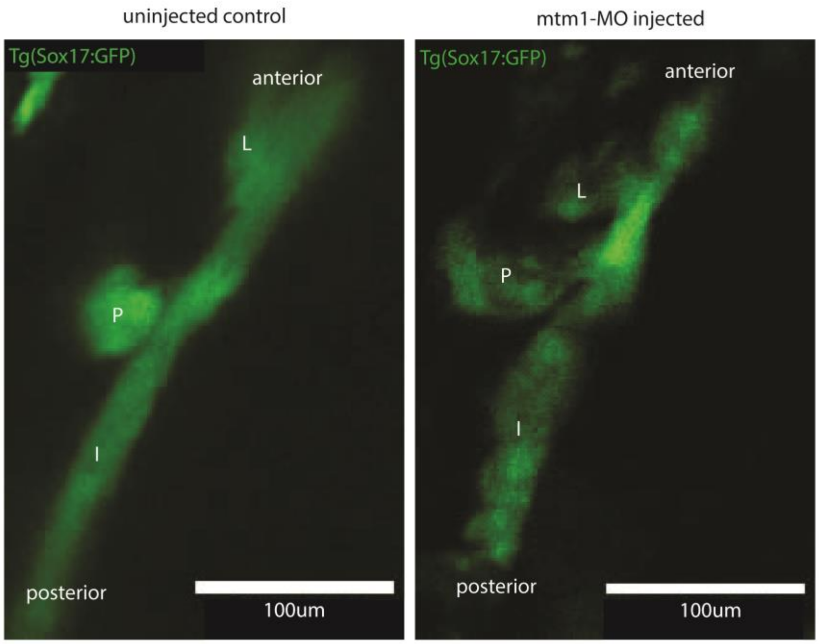

Supplemental Figure 1: Mtm1 knockdown leads to normal early endoderm development. *Tg(sox17:GFP)* fish were injected at the 1-cell stage with a morpholino targeting *mtm1*, and were imaged at 2dpf to examine the gut progenitors. *mtm1* morphants develop proper liver (L), pancreatic (P), and intestinal (I) precursors at this timepoint similar to what is seen in un-injected controls.

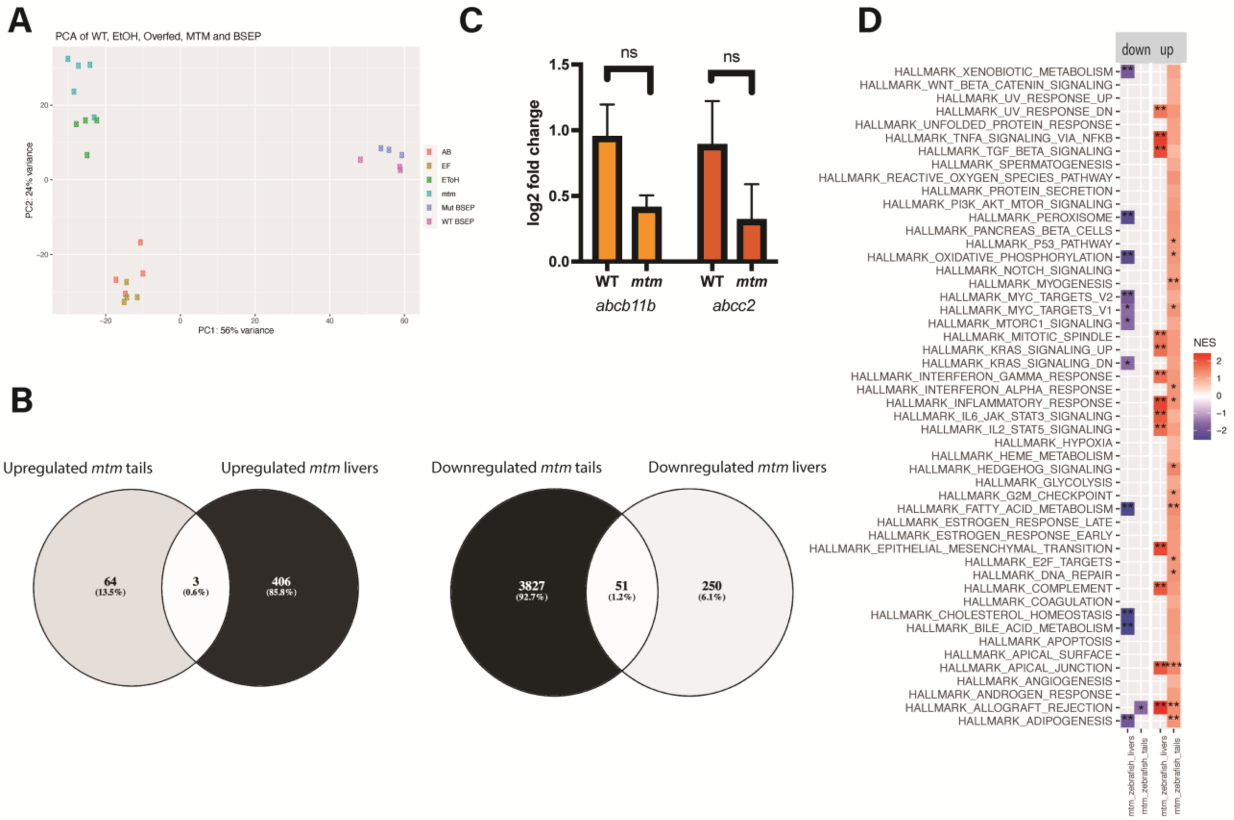

Supplemental Figure 2: Comparative RNA sequencing analyses.

(A) Principal component analysis from liver RNAseq of different disease paradigms (see Figure 5) including *abcb11b* knockout (KO) samples. The *abcb11b* KO samples cluster very distinctly from the rest, which could be due to their time point of collection (6 dpf vs 7 dpf for all other groups), the data being collected in a separate RNAseq experiment, or to differences in genetic background (AB/TU for *abcb11b* KO, AB for all remaining lines) **(B)** Venn diagrams comparing the transcriptomes of *mtm* tails (representing muscle) and *mtm* livers. There is essentially no overlap between the two groups, suggesting the transcriptional changes with *mtm1* KO in each organ are unique. **(C)** qPCR data showing RNA levels of *abcb11b* and *abcc2*, normalized to *ppib*. 3 biological replicates and 2 technical replicates were performed. **(D)** Pathway analyses comparing the *mtm* livers and tails using the fgsea (v 1.10.1 R package) analytic tool.

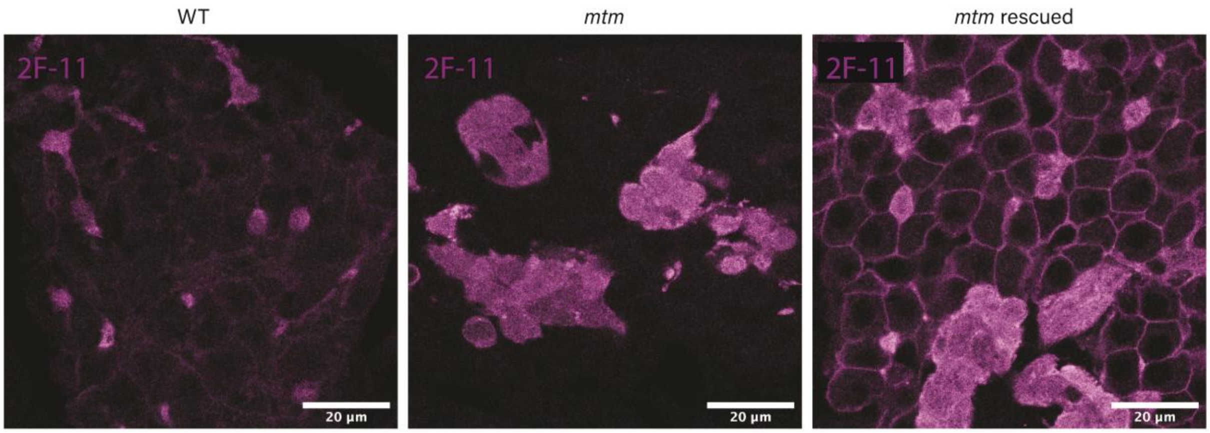

Supplemental Figure 3: *mtm1-GFP* transgene fails to rescue the bile duct phenotype. Sectioned 7 dpf zebrafish stained for 2F-11, a cholangiocyte marker. In *mtm* larvae with the *mtm1-GFP* transgene (far right), 2F-11 staining remains expanded, in addition to ectopic expression around the plasma membrane.

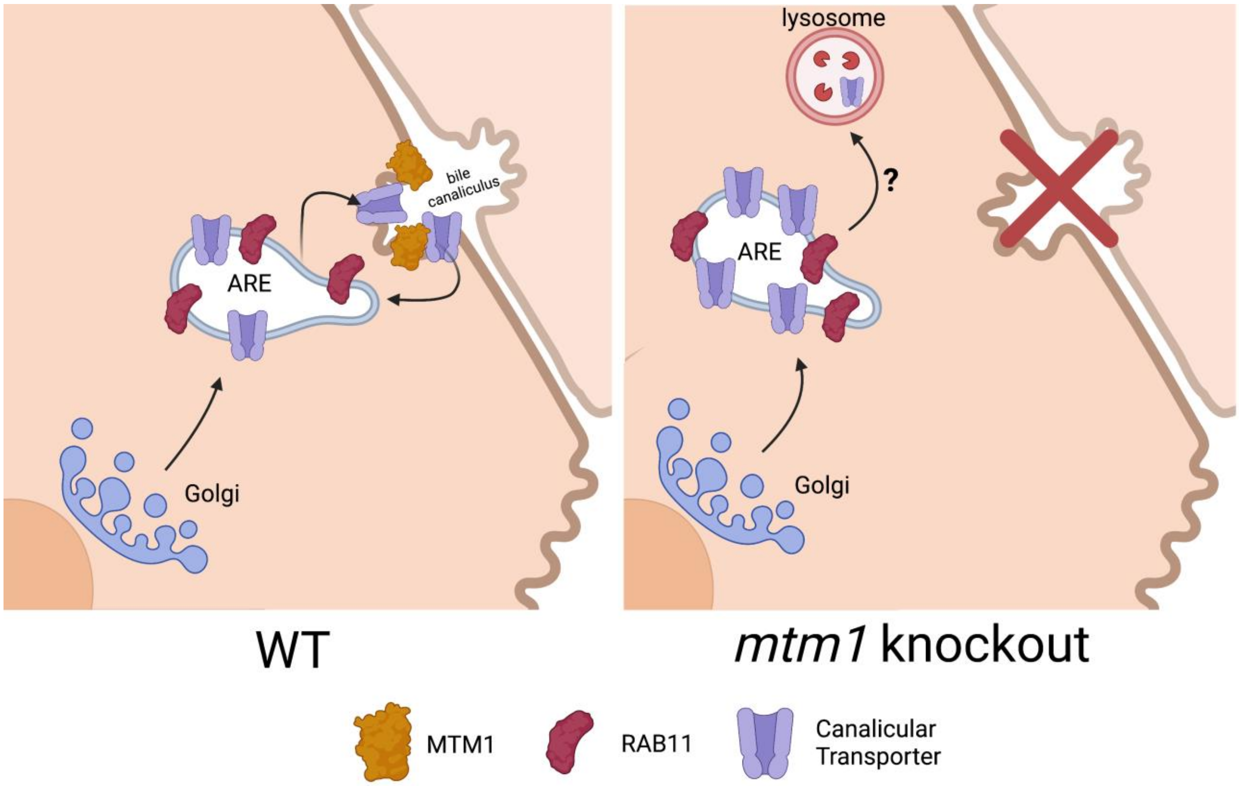

Supplementary Figure 4: Schematic showing the hypothesized role of MTM1 in regulation of canalicular protein trafficking

In wild type hepatocytes, canalicular transporters are trafficked from the Golgi apparatus into Rab11+ apical recycling endosomes (labeled ARE), from which they are trafficked to the canalicular membrane and recycled through the ARE when not needed and/or replaced with newly synthesized transporters. MTM1 appears to participate in and facilitate the regulation of this process. In the *mtm1* knockouts, the canaliculi are disturbed and there is a loss of polarity in the apical recycling endosomes. This may lead to degradation of canalicular transporters via the lysosomal compartment. This figure was generated using BioRender.
